# Supplementary material for: A superelastochromic crystal
Source: Nat Commun. 2020 Apr 14;11:1824. doi: 10.1038/s41467-020-15663-5 (PMC7156499; doi:10.1038/s41467-020-15663-5)
Supplement: Supplementary file 1 — Supplementary Information [file 41467_2020_15663_MOESM1_ESM.pdf]

Supplementary information
A superelastochromic crystal

Mutai et al.

Contents: **Page**

**Supplementary Methods**	**S3–S5**
**Supplementary Note 1. Optical properties**	**S6–S8**
**Supplementary Note 2. Crystallographic studies**	**S9–S13**
**Supplementary Note 3. Thermal analysis**	**S14–S15**
**Supplementary Note 4. Characterization of superelasticity**	**S16–S20**
**Supplementary Note 5. NMR spectra**	**S21–S22**
**Supplementary References**	**S23**

Supplementary Methods

Synthesis of 7-chloro-2-(2'-hydroxyphenyl)imidazo[1,2-*a*]pyridine (7Cl)

An acetonitrile (40 mL) solution of 2-(bromoacetyl)anisol (2.03 g, 8.86 mmol), 4-chloro-2-amino-pyridine (1.14 g, 8.86 mmol) and $NaHCO_3$ (1.55 g, 18.45 mmol) was refluxed for 20 h. After filtered off insoluble solid, the filtrate was evaporated and the residue was applied to a silica gel column ($CHCl_3$/ethyl acetate = 100:0 to 40:1) to afforded 7-chloro-2-(2'-methoxyphenyl)imidazo[1,2-*a*]pyridine (1.69 g, 74 %, m.p. 138–139°C). Then a cooled solution (0°C) of well-dried 7-chloro-2-(2'-methoxyphenyl)imidazo[1,2-*a*]pyridine (1.51 g, 5.84 mmol) in anhydrous dichloromethane was dropwise added a dichloromethane solution (1.0 mol/L) of boron tribromide (18 mL, 3.1 eq.). The reaction mixture was allowed to reach room temperature and further stirred for 3 h. A saturated aqueous $NaHCO_3$ was slowly added with stirring, and then separated with water and chloroform. The organic layer was washed with water and brine, and then dried over Na_2SO_4. The organic layer was evaporated to give crude product. Purification by recrystallization from ethanol (0.80 g, 56 %). M. p.: 196–197 °C. ^{1}H NMR ($CDCl_3$, 400 MHz) δ: 12.38 (1H, s, OH), 8.05 (1H, dd, J = 4.5, 0.5 Hz, 5-H), 7.81 (1H, s, 3-H), 7.59 (1H, sd, J = 1.3 Hz, 8-H), 7.54 (1H, dd, J = 4.7, 1.0 Hz, 6'-H), 7.23 (1H, ddd, J = 5.0, 4.5, 1.0 Hz, 4'-H), 7.02 (1H, dd, J = 5.0, 1.0 Hz, 3'-H), 6.87 (1H, ddd, J = 5.0, 4.5, 1.0 Hz, 5'-H), 6.84 (1H, dd, J = 4.3, 1.0 Hz, 6-H) (Supplementary Fig. 18). ^{13}C-NMR ($CDCl_3$, 100 MHz) δ: 157.3, 146.3, 143.4, 131.7, 130.1, 125.9, 125.6, 119.2, 117.9, 115.8, 115.8, 114.9, 106.9 (Supplementary Fig. 19). Anal. Calcd for $C_{13}H_9ClN_2O$: C,65.00; H,4.29; N,10.83%. Found: C,64.86; H,4.26; N,10.89%.

Crystal preparation

Single crystals of **7Cl** were prepared by recrystallization from ethanol solutions, affording two polymorphic crystals: YG and YO. Single crystals in the YG form were selectively obtained from a **7Cl** solution in 1:1 mixture of THF and toluene at r.t.

Microscope observations

An optical microscope (SZ61, Olympus Co.) equipped with polarizing plates and a digital camera was used to record mechanical deformation of crystals using tweezers.

Differential scanning calorimetry (DSC) measurements

DSC measurements of **7Cl** crystals were carried out using a DSC-60 (Shimadzu Co.) and DSC 7020 (Hitachi High-Technologies Co.) instruments under a nitrogen gas flow (65 ml min^{-1}). Crystals in YG and YO form with the size of a few hundreds of micrometers for the measurements were prepared by slow evaporation of a **7Cl** solution in 1:1 mixture of THF and toluene and in ethanol, respectively, at r.t. Experimental conditions are summarized in Supplementary Table 1.

Supplementary Table 1. DSC measurement conditions.

Crystal	Weight / mg	Scanning rate / °C min^{-1}	Temperature range
YG^{a}	2.78	6	30°C –220°C
YG^{b}	4.79	5	-140°C –150°C
YG^{b}	4.79	0.5	180°C –215°C
YO^{a}	3.00	6	30°C –220°C
YO^{b}	2.38	5	-140°C –150°C
YO^{b}	2.38	0.5	180°C –215°C

^{a} Measurements were taken on the DSC-60 (Shimadzu Co.) and ^{b} DSC 7020 (Hitachi High-Technologies Co.) instruments under a nitrogen gas flow (65 ml min^{-1}).

In-situ solid-state fluorescence spectroscopy

The fluorescence spectra, images, and movies of the solid samples were acquired using an Olympus BX51 optical microscope equipped with an Ocean Optics USB4000 spectrometer and a KEYENCE VB-7010 cooled CCD camera. The samples were irradiated with light guided from a high-pressure Hg lamp via a 330–380 nm bandpass filter.

Single crystal X-ray structural analysis

A mechanically deformed YG (coexisting state of α_{YG} and β_{YO} domains) and YO crystals (coexisting state of α_{YO} and β_{YG} domains) were prepared in addition to as-prepared **7Cl** single crystals in YG and YO form. To avoid spontaneous dissipation of a β_{YO} domain, the mechanically deformed YG crystal was partially cleaved at the α_{YG}//β_{YO} interfaces. Single-crystal X-ray diffraction measurements of the crystals were performed at 298 K (25 °C) on a with a CMOS detector (Bruker Photon III C14) with a nitrogen-flow temperature controller using a rotating anode X-ray source (MoKα radiation (λ = 0.71073 Å)). Multi-scan absorption corrections were applied using the SADABS program. The structure was solved by intrinsic phasing methods (SHELXT-2014/5) and refined by full-matrix least-squares calculations on F^2 (SHELXL-2016/6). Non-hydrogen atoms were refined anisotropically; hydrogen atoms were fixed at calculated positions by riding model approximation. With respect to the mechanically deformed crystals, X-ray diffraction patterns were obtained around the interface of α_{YG}//β_{YO} and α_{YO}//β_{YG} and they were analyzed as twin. Crystal face indexing was carried out using APEX III Ver.2016.1-0 program package with a twin resolution program (Supplementary Figs. 4 and 5).

Force measurements

Shear tests were carried out on a universal testing machine. A crystal fixed on a glass base was sheared by a glass jig and observed under an optical microscope equipped with polarizing plates. Light sources: LA-HDF5010 (90 W, HAYASHI-REPIC CO., LTD.) for VIS light, HLV-24UV365-4WPCLTL (365 nm, 0.7 A/3.3 W, CCS Inc.) connected to PD3-5024-4-EI(A) (CCS Inc.) for UV light. The experimental information and schematic representation of the setup is shown in Supplementary Table 2 and Supplementary Fig. 1, respectively.

Supplementary Table 2 Experimental information of shear tests on **7Cl** crystals.

Specimen	Conditions		Crystal dimension	
	temperature/°C	light	width/μm	thickness/μm
YG-1	20	PW	246	20
YG-2	20	Dark	370	23
YG-3	20	UV	470	20
YG-4	0	PW	180	20
YG-5	100	PW	470	20

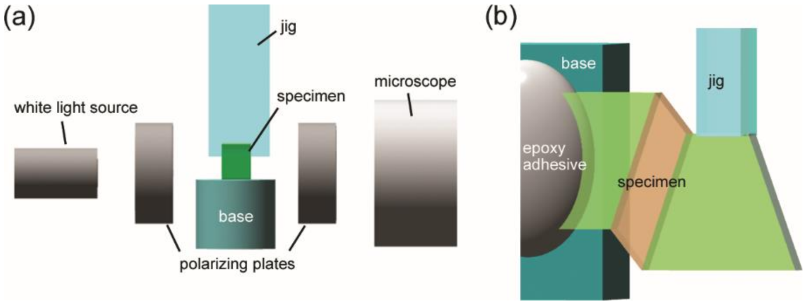

Supplementary Fig. 1 Experimental setup of shear tests. Setups for (a) microscope observation and (b) shearing a crystal.

Supplementary Note 1. Optical properties

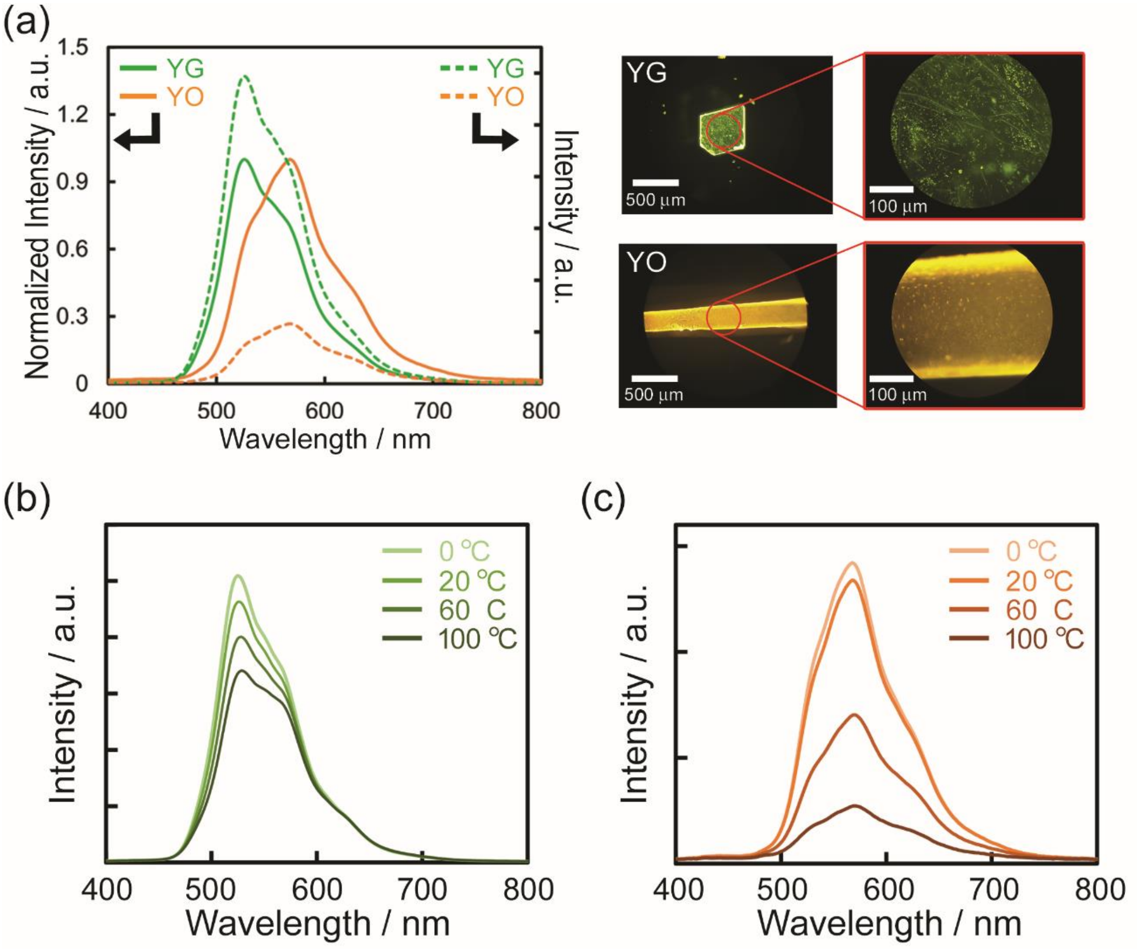

Supplementary Fig. 2 Fluorescence spectroscopy. (a) Solid-state emission spectra of a YG crystal (yellow green dotted line) and a YO crystal (orange dotted line) excited by UV light (330–380 nm) at RT and photos of YG and YO crystals for the measurements. Their normalized spectra are also shown by solid lines. (b) Temperature-dependent fluorescence spectroscopy of YG and (c) YO crystals from 0 °C–100 °C excited by UV light (330–380 nm). The spectra were measured by an Olympus BX51 optical microscope equipped with an Ocean Optics USB4000 spectrometer.

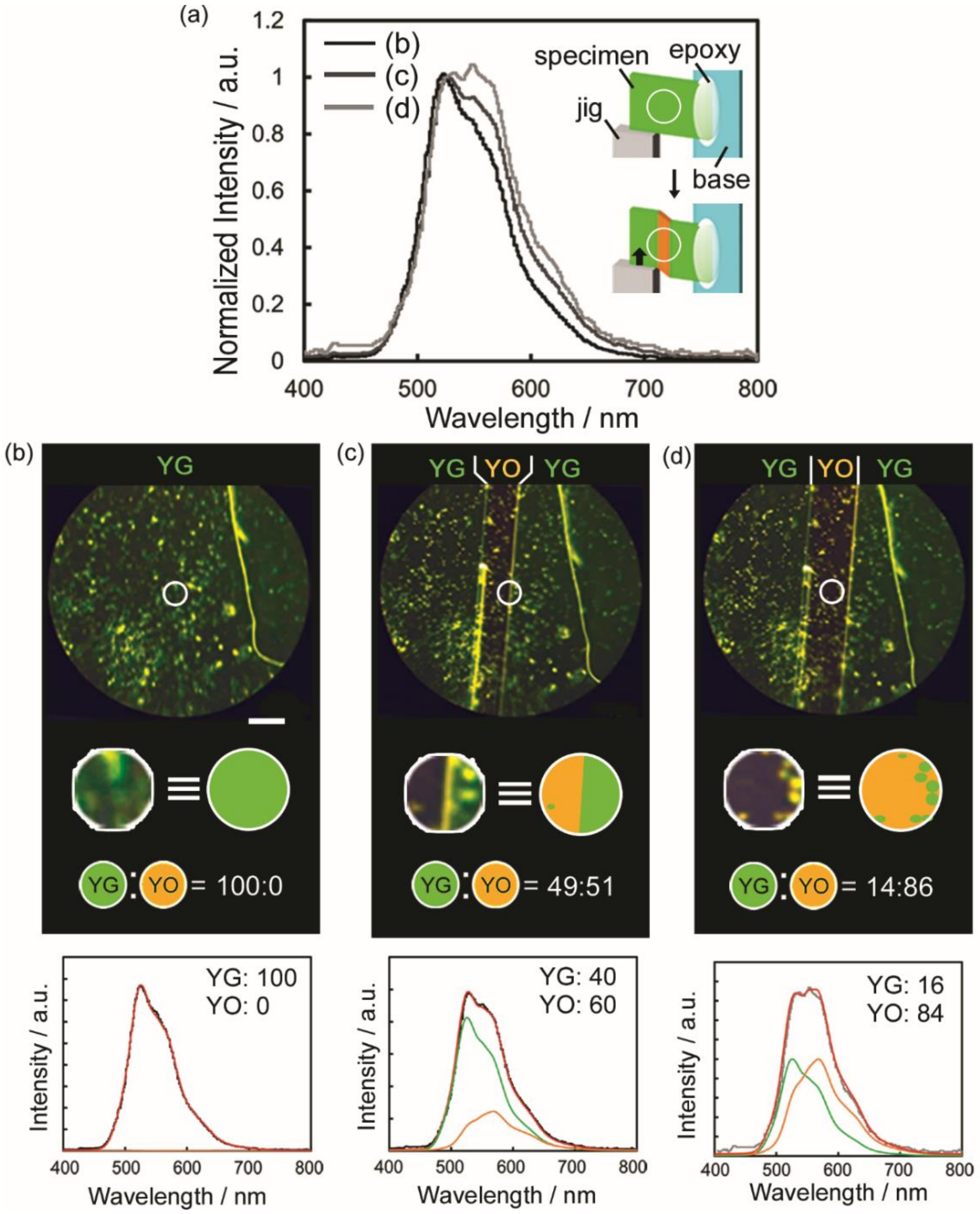

Supplementary Fig. 3 In situ fluorescence spectroscopy. (a) Schematic drawing of the experimental set up and fluorescence spectra showing changes in fluorescence intensity of a YG crystal from the initial state to superelastically deformed state under UV light (330–380 nm) at r.t. (normalized by the intensity at 525 nm). (b) Snapshots (top) and fluorescence spectra (bottom) of the crystal during superelastic deformation in the initial state and (c), (d) superelastically deformed states. (Scale bar, 50 μm) White lines and circles represent position of $\alpha_{YG}//\alpha_{YO}$ interfaces and detection area of fluorescence emission by an Olympus BX51 optical microscope equipped with an Ocean Optics USB4000 spectrometer. The mixing ratio of α_{YG}: α_{YO} in each deformation state was calculated based on image analysis and wave separation analysis under consideration of the fluorescence intensity ratio (YG:YO = 1:0.195). Black, dark gray, and light gray lines represent spectra of a **7Cl** crystal in the state of (b), (c), (d). These spectra were reproduced from those of YG (yellow green line) and YO (orange line) crystals and shown by red lines.

Pure YG and YO crystals show fluorescent spectra with a peak top/shoulder at 525 nm/568 nm and 568 nm/531 nm, respectively (Supplementary Fig. 2a). By proceeding conversion of an α_{YG} domain into α_{YO} domain of a YG crystal, the pure YG fluorescent spectrum changed into a YG-YO mixed one (Supplementary Fig. 3a). The mixing ratios of α_{YG}:α_{YO} calculated based on wave separation analysis and image analysis in the detection area of fluorescence (Supplementary Fig. 3b–d white circles) varied as 100:0 → 40:60 → 16:84 and 100:0 → 49:51 → 14:86, respectively (Supplementary Fig. 3b–d). Although the calculated ratio values are not completely consistent with each other most likely due to strong fluorescent emission from crystal edges, the correlating change of fluorescent spectra with the α_{YG}:α_{YO} domain ratio clearly indicates possible applicability of YG crystals as a detector of mechanical stress by superelastical fluorescence change.

Supplementary Note 2. Crystallographic studies

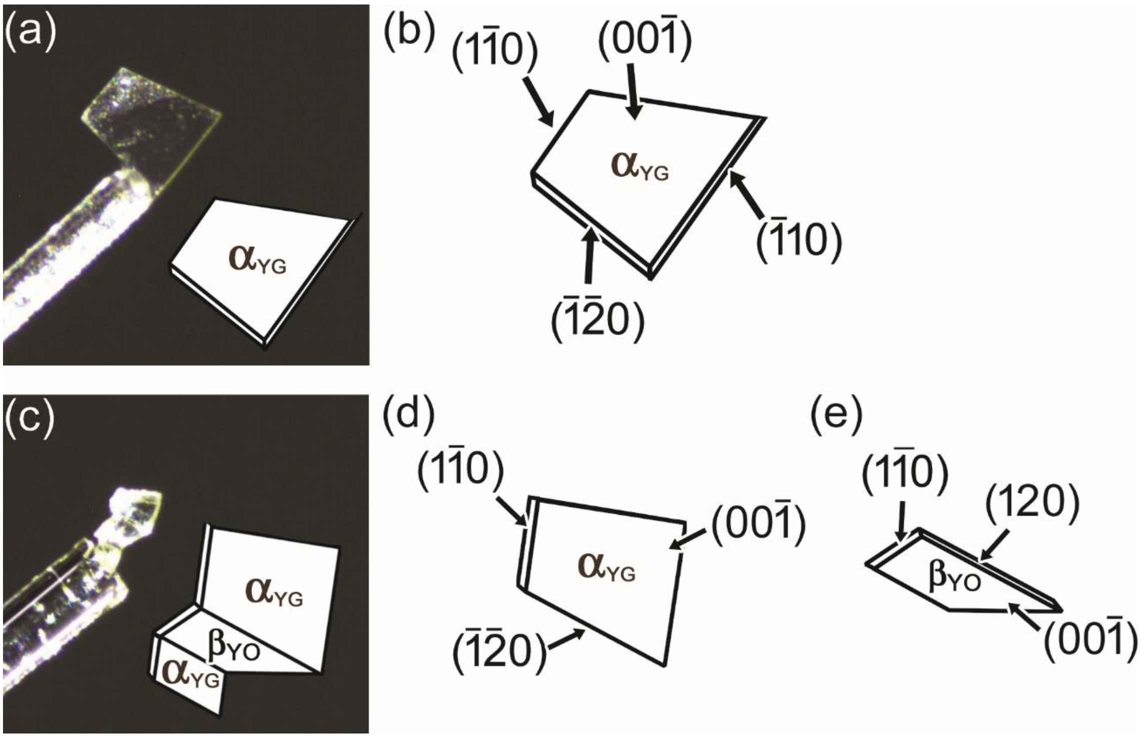

Supplementary Fig. S4 Crystallogphic studies of YG crystals. (a) A photo of as-prepared YG crystal and (b) its phase indexing. (c) A photo of a mechanically deformed YG crystal and its phase indexing of (d) mother (α_{YG}) domain and (e) a shear-induced daughter (β_{YO}) domain through the superelastic deformation.

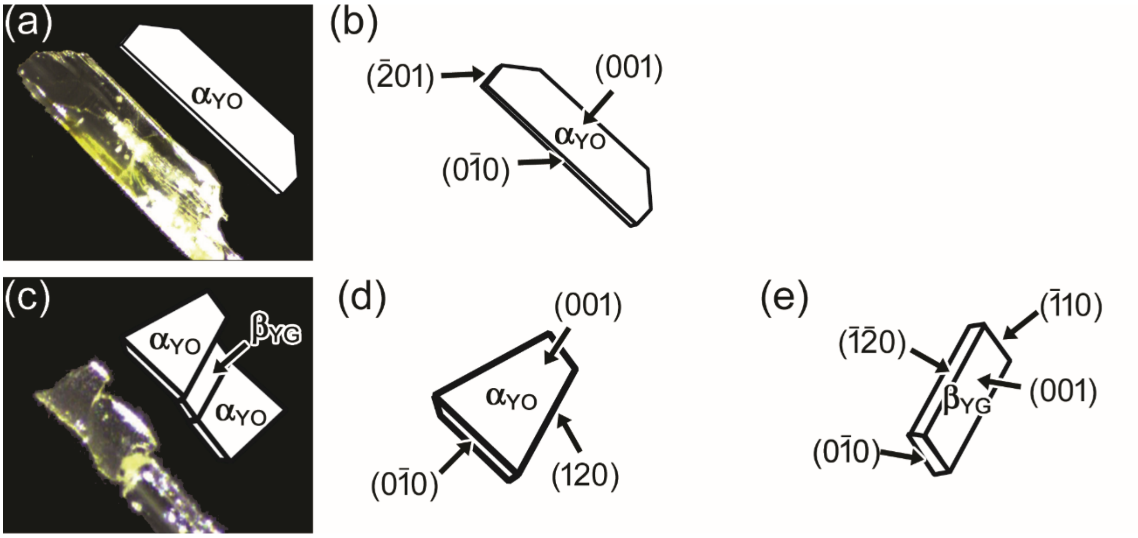

Supplementary Fig. 5 Crystallographic studies of YO crystals. (a) A photo of an as-prepared YO crystal and (b) its phase indexing. (c) A photo of a mechanically deformed YO crystal and its phase indexing of (d) mother (α_{YO}) domain and (e) a shear-induced daughter (β_{YG}) domain through the superelastic deformation.

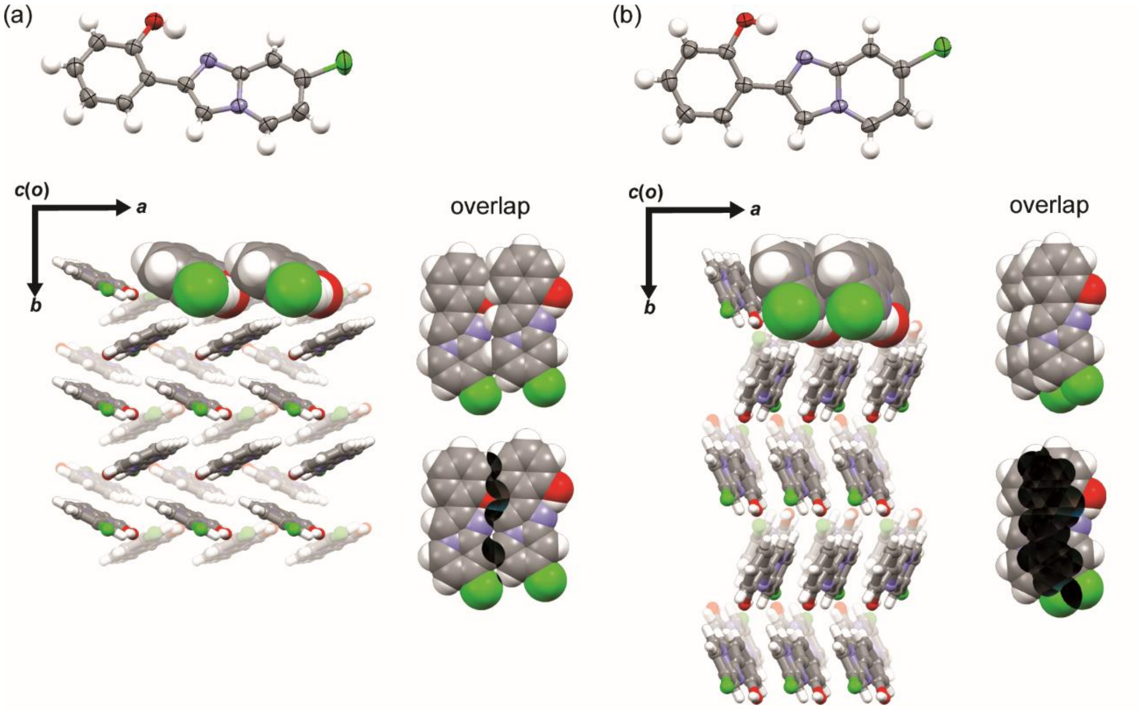

Supplementary Fig. 6 Thermal ellipsoids (50% probability level), packing diagrams, and overlaps of **7Cl** moleculesin a (a) YG crystal and (b) YO crystal.

Supplementary Table 3 Crystallographic data of YG crystals: as-prepared and mechanically deformed crystals.

Entry	YG (as-prepared)	YG (mechanically deformed)	
Domain	α_{YG}	α_{YG} (Mother)	β_{YO} (Daughter)
T /K	298	298	298
Empirical formula	$C_{13}H_9ClN_2O$	$C_{13}H_9ClN_2O$	$C_{13}H_9ClN_2O$
Crystal size /mm^3	0.32x0.26x0.02	0.20x0.11x0.08	0.20x0.07x0.08
M	244.67	244.67	244.67
Crystal system	Monoclinic	Monoclinic	Monoclinic
Space group	$P2_1/n$	-	-
a /Å	5.8359(12)	5.8366(17)	3.86
b /Å	7.5055(13)	7.506(2)	11.31
c /Å	25.145(4)	25.149(8)	24.72
α /°	90	90	90
β /°	90.138(7)	90.137(9)	93.48
γ /°	90	90	90
V /Å^3	1101.4(3)	1101.7(6)	1077
Z	4	-	-
D_{calcd} /Mg m^{-3}	1.476	-	-
μ(Mo Kα) / mm^{-1}	0.329	-	-
Reflections collected	12616	-	-
Independent reflections (R_{int})	1938 (0.0357)	-	-
Goodness of fit	1.038	-	-
R_1($I > 2\sigma$ (all data))	0.0381 (0.0552)	-	-
$_wR_2$($I > 2\sigma$ (all data))	0.0961 (0.1114)	-	-
Largest diff. peak (hole) /eÅ^3	0.194 (-0.217)	-	-
CCDC No.	1969297	-	-

Supplementary Table 4 Crystallographic data of YO crystals: as-prepared and mechanically deformed crystals.

Entry	YO (as-prepared)	YO (mechanically deformed)	
Domain	α_{YO}	α_{YO} (Mother)	β_{YG} (Daughter)
T /K	298	298	298
Empirical formula	$C_{13}H_9ClN_2O$	$C_{13}H_9ClN_2O$	$C_{13}H_9ClN_2O$
Crystal size /mm^3	1.70x0.35x0.07	0.21x0.16x0.03	0.19x0.08x0.03
M	244.67	244.67	244.67
Crystal system	Monoclinic	Monoclinic	Monoclinic
Space group	$P2_1/n$	-	-
a /Å	3.8600(2)	3.89	5.84
b /Å	11.3300(6)	11.40	7.51
c /Å	24.8812(14)	25.00	25.18
α /°	90	90	90
β /°	93.053(2)	92.90	90.15
γ /°	90	90	90
V /Å^3	1086.60(10)	1108	1103
Z	4	-	-
D_{calcd} /Mg m^{-3}	1.496	-	-
μ(Mo Kα) / mm^{-1}	0.333	-	-
Reflections collected	11821	-	-
Independent reflections (R_{int})	1931 (0.0463)	-	-
Goodness of fit	0.879	-	-
R_1($I > 2\sigma$ (all data))	0.0455 (0.0535)	-	-
$_wR_2$($I > 2\sigma$ (all data))	0.1366 (0.1474)	-	-
Largest diff. peak (hole) /eÅ^3	0.378 (-0.279)	-	-
CCDC No.	1969298	-	-

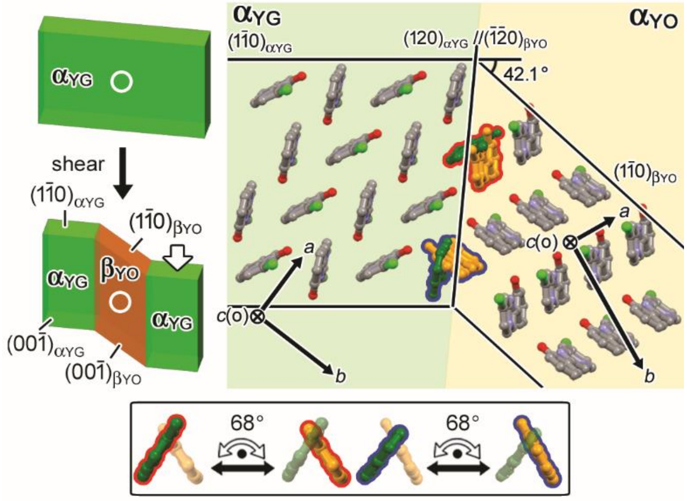

Supplementary Fig. 7 Crystal structure of a superelastically deformed 7Cl crystal. Schematic representation of superelastic deformation and estimated molecular correspondence at the $\alpha_{YG}//\beta_{YO}$ interface in a deformed **7Cl** crystal based on X-ray results. White circles indicate a fluorescence detection area of in-situ fluorescence spectroscopy in Supplementary Fig. 3. Inset represents possible molecular movement during the interconversion between α_{YG} and β_{YO} domains. Molecules at the interface are colored in dark green (α_{YG}) and orange (β_{YO}).

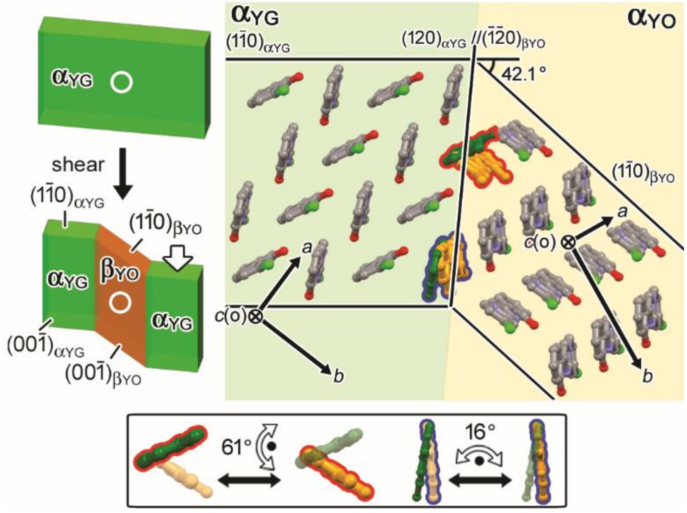

Supplementary Fig. 8 Crystal structure of a superelastically deformed 7Cl crystal. Schematic representation of superelastic deformation and estimated molecular correspondence at the $\alpha_{YG}//\beta_{YO}$ interface in a deformed **7Cl** crystal based on X-ray results. White circles indicate a fluorescence detection area of in-situ fluorescence spectroscopy in Supplementary Fig. 3. Inset represents possible molecular movement during the interconversion between α_{YG} and β_{YO} domains. Molecules at the interface are colored in dark green (α_{YG}) and orange (β_{YO}).

Supplementary Note 3. Thermal analysis

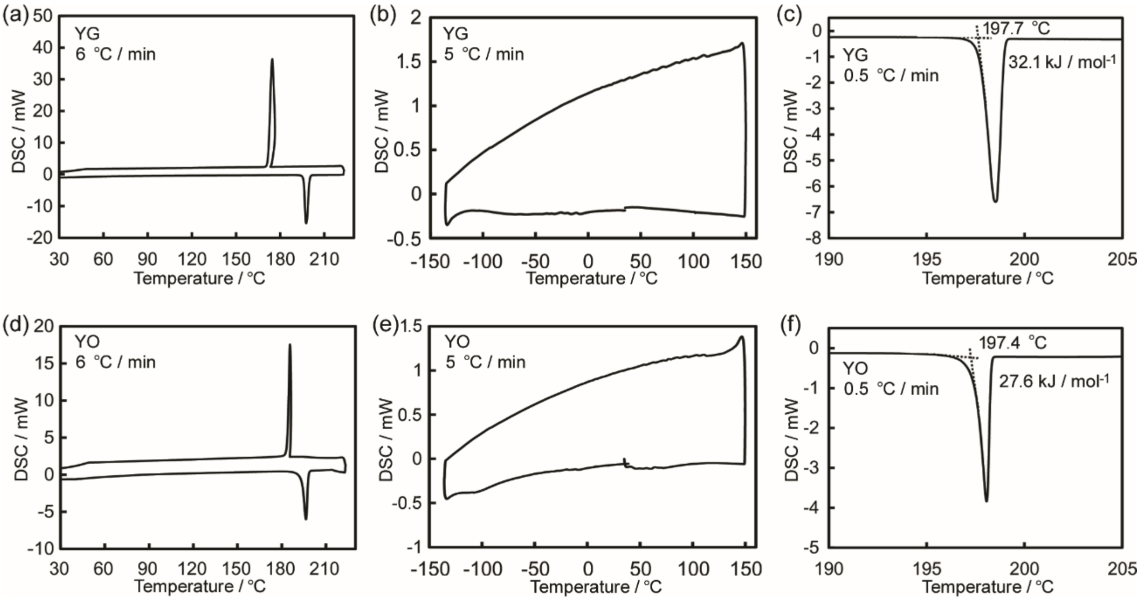

Supplementary Fig. 9 Differential scanning calorimetry measurements. Charts (a)–(c) show a YG crystal and charts (d)–(f) show a YO crystal. The melting points of the YG and YO crystals were estimated at 197.7°C and 197.4°C, respectively.

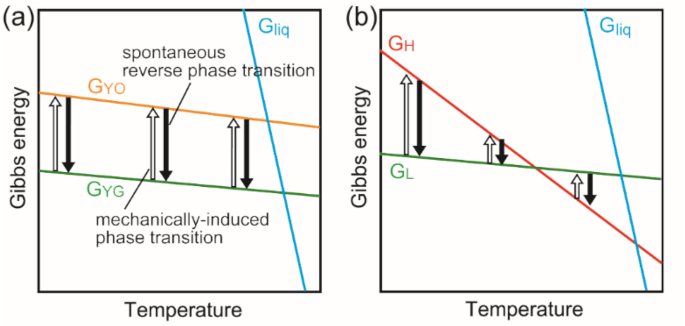

Supplementary Fig. 10 Schematic representation of estimated Gibbs energy. (a) Gibbs energy diagram of a YG crystal (G_{YG}), YO crystal (G_{YO}), and liquid phase (G_{liq}). (b) Gibbs energy diagram of a crystal showing superelasticity by mechanically-induced phase transition (high-temperature phase: G_H, low-temperature phase: G_L, liquid phase: G_{liq}).

The monotropic nature of a **7Cl** crystal was suggested by DSC measurements on YG and YO crystals which shows no thermally-induced phase transition between YG and YO crystals (Supplementary Fig. 9). In addition, Gibbs energy gap between YG and YO crystals is expected to be almost constant (Supplementary Fig. 10a) because shear stress for the mechanically-induced phase transition at 0 °C, 20 °C, and 100 °C is comparable to each other (Supplementary Figs. 12, 13, and 14). On the other hand, superelasticity based on phase transition in an enantiotropic system, e.g., terephthalamide,[1] tetra-*n*-butylphosphonium tetraphenylborate,[2] and aliphatic acids[3] has shear stress showing large temperature dependence which reflects rapid changes in the Gibbs energy gap between a high and low temperature phases (Supplementary Fig. 10b).

Supplementary Table 5 Thermodynamic parameters obtained using DSC measurements for **7Cl** crystals.

Crystal	Melting point/°C	ΔH/kJ mol^{-1}	ΔS/JK^{-1} mol^{-1}
YG	197.7	32.1	68.1
YO	197.4	27.6	58.8

Supplementary Note 4. Characterization of superelasticity

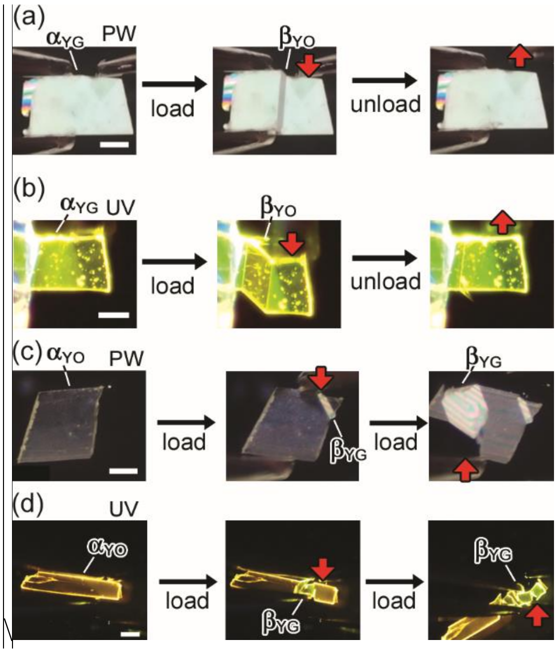

Supplementary Fig. 11 Deformation of YG and YO crystals. (a) Snapshots of deformation of a YG crystal by mechanically induced phase transition from YG to YO under PW light (scale bar, 200 μm) and (d) under UV light irradiation (scale bar, 100 μm). (b) Snapshots of deformation of a YO crystal by mechanically induced phase transition from YO to YG under PW light (scale bar, 100 μm) and (d) under UV light irradiation (scale bar, 300 μm).

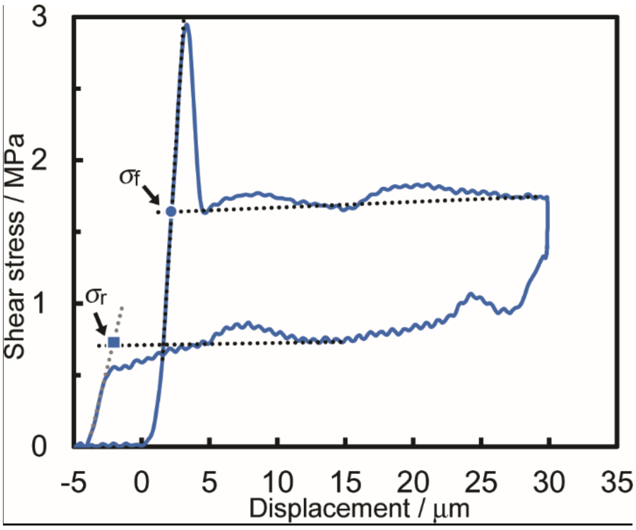

Supplementary Fig. 12 Evaluation of temperature dependence. A stress-displacement curve of a YG crystal under PW light at 0 °C.

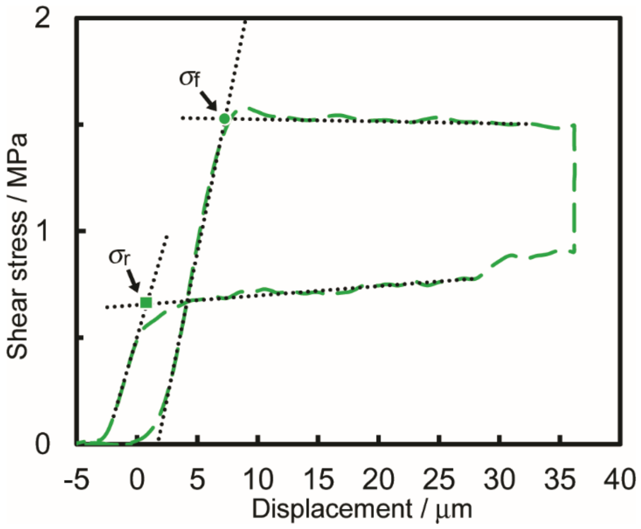

Supplementary Fig. 13 Evaluation of temperature dependence. A stress-displacement curve of a YG crystal under PW light at r.t.

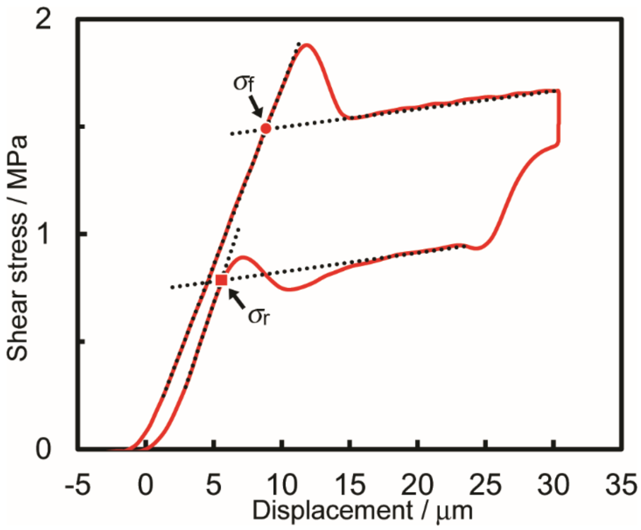

Supplementary Fig. 14 Evaluation of temperature dependence. A stress-displacement curve of a YG crystal under PW light at 100 °C.

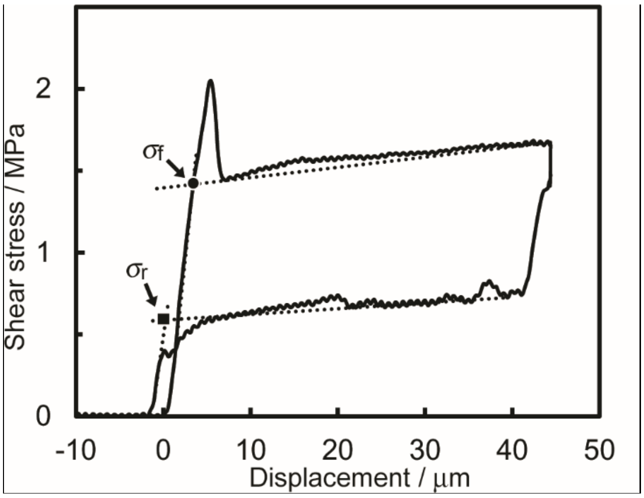

Supplementary Fig. 15 Evaluation of light dependence. A stress-displacement curve of a YG crystal in the dark at r.t.

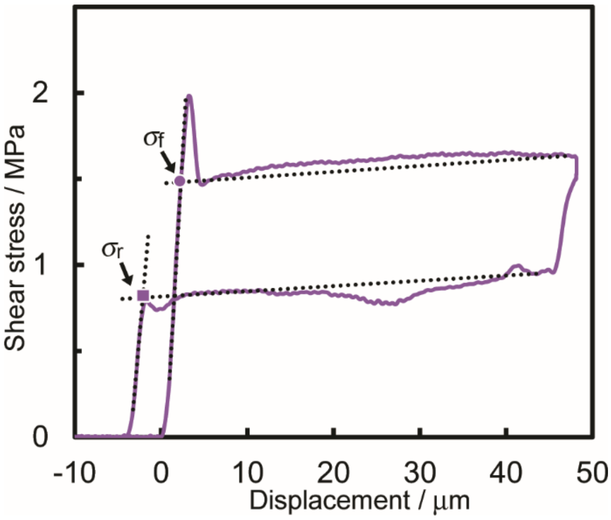

Supplementary Fig. 16 Evaluation of light dependence. A stress-displacement curve of a YG crystal under UV light at r.t.

Supplementary Table 6 Mechanical parameters of YG crystals under various condition.

Light	Dark	PW	UV	PW	PW
Temperature (°C)	20	20	20	0	100
σ_f (MPa)[a]	1.417	1.527	1.481	1.639	1.488
σ_r (MPa)[b]	0.588	0.657	0.810	0.706	0.785
σ_c (MPa)[c]	1.002	1.092	1.146	1.173	1.136
E_s (kJ m^{-3})[d]	495.7	553.6	682.5	595.1	661.4
E_d (kJ m^{-3})[e]	697.9	733.0	565.8	785.7	592.3
η[f]	0.415	0.430	0.547	0.431	0.528
χ[g]	0.493	0.505	0.593	0.506	0.580

[a,b]Effective shear stress for forward (σ_f) and reverse (σ_r) superelastic deformation. [c]Chemical shear: σ_c=(σ_f+σ_r)/2. [d–g]Energy storage density: E_s=W_{out}/V, dissipated energy density: E_d=W_{in}/V, energy storage efficiency: η=W_{out}/W_{in}, and [g]superelastic index: χ= E_s/σ_c, respectively, where W_{out}, W_{in}, and V represent output work, input work, and volume of a deformed region, respectively, during superelastic deformation of YG crystals.

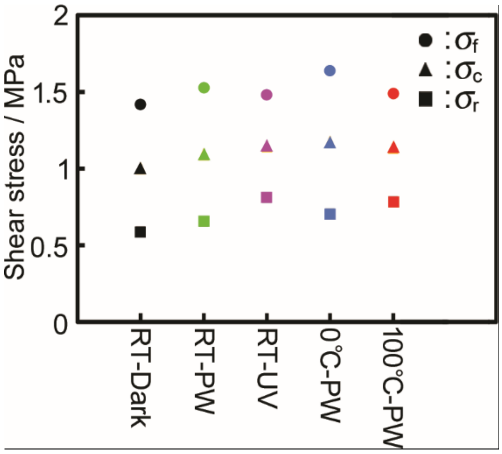

Supplementary Fig. 17 Environment dependence of superelasticity. Effective shear stress values for forward and reverse deformation (σ_f: filled circle, and σ_r: filled square) and chemical shear (σ_c = (σ_f + σ_r)/2: filled triangle) during superelastic deformation of crystals of YG under various conditions: without light at r.t. (RT-Dark), under PW light at r.t. (RT-PW), under UV light at r.t. (RT-UV), under PW light at 0 °C (0 °C-PW), and under PW light at 100 °C (100 °C-PW).

Supplementary Table 7 Mechanical characteristics of a YG crystal and representative organosuperelastic crystals.

	Telephthalamide[a]	3,5-Difluorobenzoic acid[b]	YG
σ_f (MPa)[c]	0.496	0.07	1.527
σ_r (MPa)[d]	0.459	0.013	0.657
σ_c (MPa)[e]	0.478	0.04	1.092
E_s (kJ m^{-3})[f]	62.0	11.9	553.6
η[g]	0.925	0.178	0.430
χ[h]	0.129	0.263	0.505

[a]See ref. 1. [b]See ref. 4. [c,d]Effective shear stress for forward (σ_f) and reverse (σ_r) superelastic deformation. [e–h]Chemical shear: $\sigma_c=(\sigma_f+\sigma_r)/2$, energy storage density: $E_s=W_{out}/V$, energy storage efficiency: $\eta=W_{out}/W_{in}$, superelastic index: $\chi= E_s/\sigma_c$, respectively, where W_{out}, W_{in}, and V represent output work, input work, and volume of a deformed region, respectively, during superelastic deformation.

Supplementary Note 5. NMR spectra

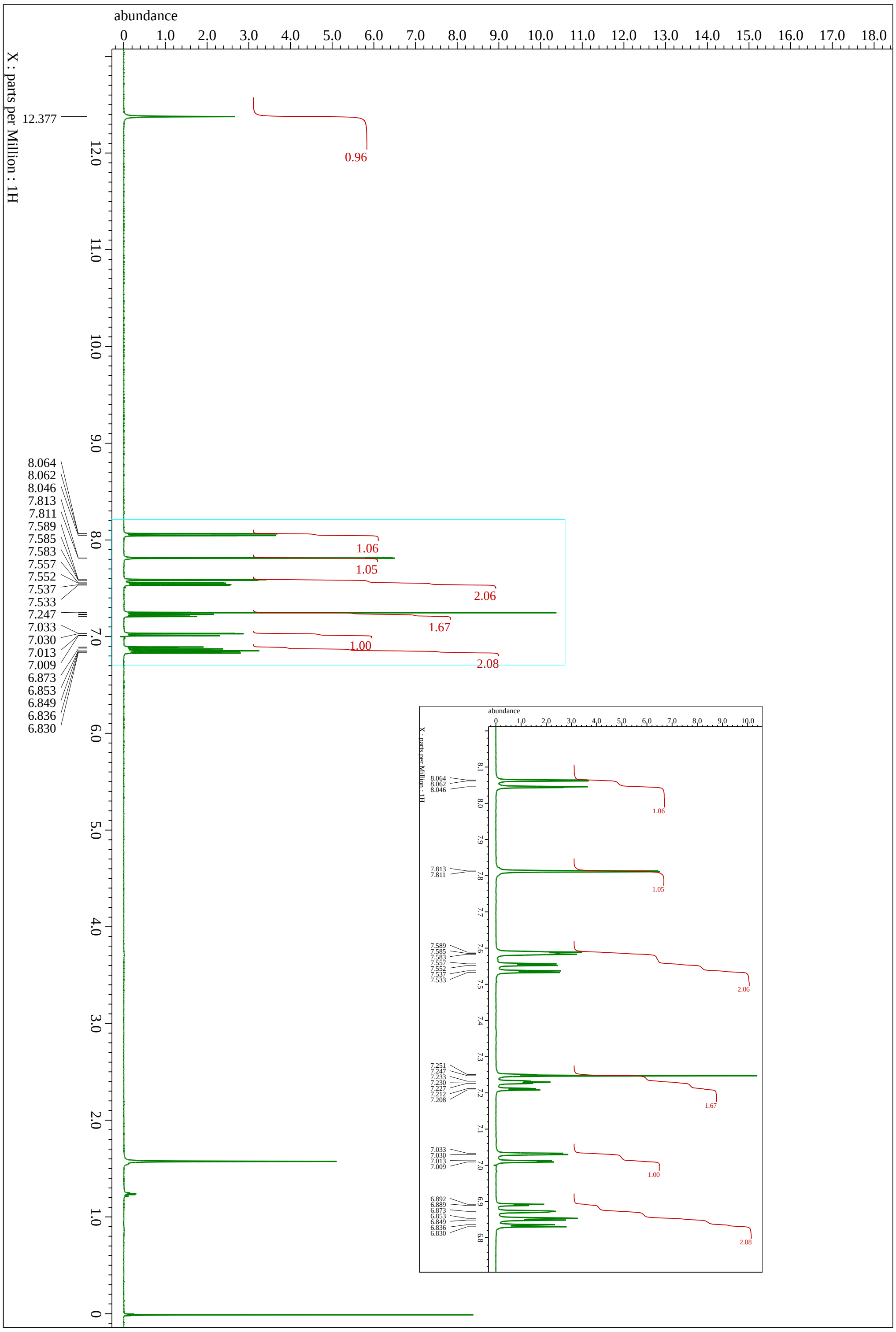

Supplementary Fig. 18 ¹H NMR spectrum (CDCl₃, 400 MHz) of 7Cl.

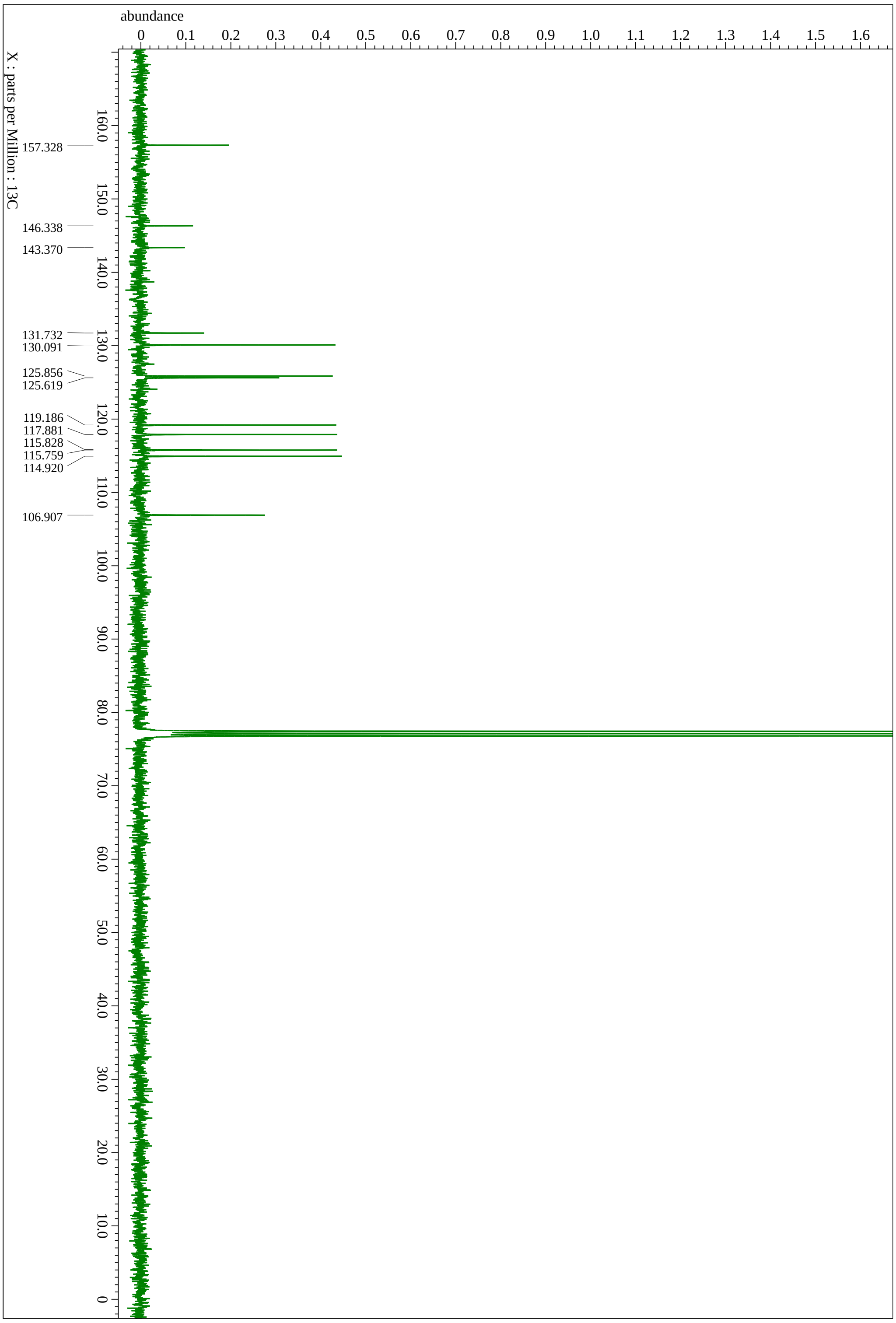

Supplementary Fig. 19 ^{13}C NMR spectrum (CDCl$_3$, 100 MHz) of 7Cl.

Supplementary References

[1]. Takamizawa, S. & Miyamoto, Y. Superelastic organic crystals. *Angew. Chem. Int. Ed.* **53**, 6970–6973 (2014).

[2]. Takamizawa, S. & Takasaki Y. Shape-memory effect in an organosuperelastic crystal. *Chemical Science* **7**, 1527–1534 (2016).

[3]. Takamizawa, S. & Takasaki Y. Versatile shape recoverability of odd-numbered saturated long-chain fatty acid crystals. *Cryst. Growth Des.* **19**(3), 1912–1920 (2019).

[4]. Takamizawa, S. & Takasaki, Y. Superelastic shape recovery of mechanically twinned 3,5-difluorobenzoic acid crystals. *Angew. Chem. Int. Ed.* **54**, 4815–4817 (2015).
