# Supplementary material for: A superelastochromic crystal
Source: Nat Commun. 2020 Apr 14;11:1824. doi: 10.1038/s41467-020-15663-5 (PMC7156499; doi:10.1038/s41467-020-15663-5)
Supplement: Supplementary file 3 — Description of Additional Supplementary Files [file 41467_2020_15663_MOESM3_ESM.pdf]

Description of Additional Supplementary Files

File Name: Supplementary Movie 1
Description: Mechanically-induced phase transition in a YG and YO crystal under ultraviolet (365 nm) light (UV, the first half) and under polarized white light (PW, the last half) at room temperature (RT).

File Name: Supplementary Movie 2
Description: Superelastic behavior of a YG crystal under polarized white light (PW, the first half) and under ultraviolet (365 nm) light (UV, the last half) at room temperature (RT).
